# P100 Wave Latency and Amplitude in Visual Evoked Potential Records in Different Visual Quadrants of Normal Individuals

**DOI:** 10.18502/jovr.v18i2.13184

**Published:** 2023-04-19

**Authors:** Leila Mirzaee Saba, Hassan Hashemi, Ebrahim Jafarzadehpour, Ali Mirzajani, Abbasali Yekta, Abolfazl Jafarzadehpour, Arghavan Zarei, Payam Nabovati, Mehdi Khabazkhoob

**Affiliations:** ^1^Noor Research Center for Ophthalmic Epidemiology, Noor Eye Hospital, Tehran, Iran; ^2^Noor Ophthalmology Research Center, Noor Eye Hospital, Tehran, Iran; ^3^Rehabilitation Research Center, Department of Optometry, School of Rehabilitation Sciences, Iran University of Medical Sciences, Tehran, Iran; ^4^Refractive Errors Research Center, Mashhad University of Medical Sciences, Mashhad, Iran; ^5^Department of Basic Sciences, School of Nursing and Midwifery, Shahid Beheshti University of Medical Sciences, Tehran, Iran

**Keywords:** Amplitude, Latency, Normal Vision, Pattern Reversal, Visual Evoked Potential, Visual Field

## Abstract

**Purpose:**

Assessment of the pattern visual evoked potential (PVEP) responses in different areas of visual fields in individuals with normal vision.

**Methods:**

This study was conducted on 80 eyes of normal subjects aged 18–35 years. All participants underwent refraction and visual acuity examination. Visual evoked potential (VEP) responses were recorded in different areas of field. The repeated measure test was used to compare the P100 latency and amplitude of PVEP among different areas.

**Results:**

The repeated measures analysis of variance showed a statistically significant difference among different areas in terms of amplitude and latency of P100 (*P* = 0.002 and *P* < 0.001, respectively). According to the results, the highest and lowest amplitude of P100 was observed in inferior-nasal and superior areas, respectively. The highest and lowest latency of P100 was related to the temporal and inferior-nasal areas, respectively.

**Conclusion:**

This study partially revealed the details of local PVEP distribution in the visual field and there was a significant difference in the amplitude and latency of PVEP wave in different areas of the visual field.

##  INTRODUCTION

Visual field (VF) plays a significant role in clinical optometry and assessment of VF findings is important in the diagnosis and treatment of the diseases like glaucoma. ${}^{\left[1,2\right]}$ Humphrey perimetry is the gold standard for detection of visual field defects; however, it has some limitations.^[3]^ As it is a subjective test, it is largely patient dependent and the test duration may vary depending on the aptitude of the examinee. Many individuals, especially older patients, are weak on subjective tests.^[4,5,6]^ Subjective perimetry is associated with a learning curve leading to complicating interpretation of the results in new patients. As a result, the test should be performed two to three times to obtain a valid result. ${}^{\left[3,7\right]}$ Moreover, it is expected that a considerable loss of ganglion cells occurs before standard perimetry shows a visual field defect. Therefore, more sensitive tests are required because about 25–50% of optic nerve fibers may be lost before a visual field defect is actually diagnosed.^[4,8]^

Electroretinogram (ERG)^[7]^ and visual evoked potential (VEP) are two objective methods used for the measurement of visual field sensitivity.^[3,8]^ Studies show that multifocal visual evoked potential (mfVEP) is one of the most advanced technology used in the measurement of visual field sensitivity.^[9]^ Stability of the mfVEP system depends on a number of environmental factors. Since the VEP amplitude is measured within the range of microvolts, environmental factors such as noises that result from problems which occur with cortical electric changes in power supply such as voltage fluctuations may cause amplitude changes in one area of the field when presenting the stimulus. Therefore, in order to decrease the noise effect, the calculation of more comparative averages is required for an overall more accurate averaging assessment. Unfortunately, the technology used in the mfVEP software does not produce many averages because the examination is time consuming.^[3,10]^ In addition, as the mfVEP software is very complicated and costly, most centers are not equipped with the software. The present study aimed to assess the sensitivity of pattern visual evoked potential (PVEP) in different areas of visual field while considering the need for objective perimetry with the unavailability of the mfVEP software, and also considering the emphasis on clinical electrophysiology standards regarding multiple definitions of “normal” for age and race.^[10]^ To decrease the effect of noise, the averaging of 100 measurements was considered in each area. For this purpose, a stimulus was presented at each of the eight areas to measure P100 amplitude and latency in PVEP records of visually normal participants.

##  METHODS

This cross-sectional study was conducted to assess the P100 amplitude and latency of PVEP in different visual field areas in visually normal individuals in the Ophthalmic Electrophysiology Clinic of the School of Rehabilitation, Iran University of Medical Sciences. The participants were selected from eligible individuals attending the clinic who were willing to join the study and signed an informed consent form through convenient sampling. The inclusion criteria were as follows: (1) age 18–35 years, (2) corrected distance visual acuity of 20/20 or better in both eyes (best visual acuity 20/20 with or without correction), (3) myopia < –3 diopters (D) or astigmatism < 2D,^[11]^ and (4) lack of any systemic diseases that might affect PVEP results.

Based on the values obtained from the articles, the mentioned standard deviation reported to be around 4.7.^[12]^ Taking into account the value of d equal to 2 and the confidence level of 95% and z equal to 1.96, based on the following equation the calculated number of samples would be 21; we however examined 40 people in this study. 


$$N=\frac{Z^{2}\delta^{2}}{d^{2}}.$$

The objective of the study was explained to the participants. First, visual acuity was tested with a Snellen chart and then objective refraction was measured using a Heine retinoscope (Heine, Germany) and the Huvitz HRK-8000 autorefractometer (Huvitz, South Korea). Subjective refraction responses were also evaluated. Eyes were examined with slit lamp and ophthalmoscope for eye pathology determination. In the next stage, the P100 amplitude and latency of PVEP were recorded in mesopic conditions.

##  PVEP

**Figure 1:**
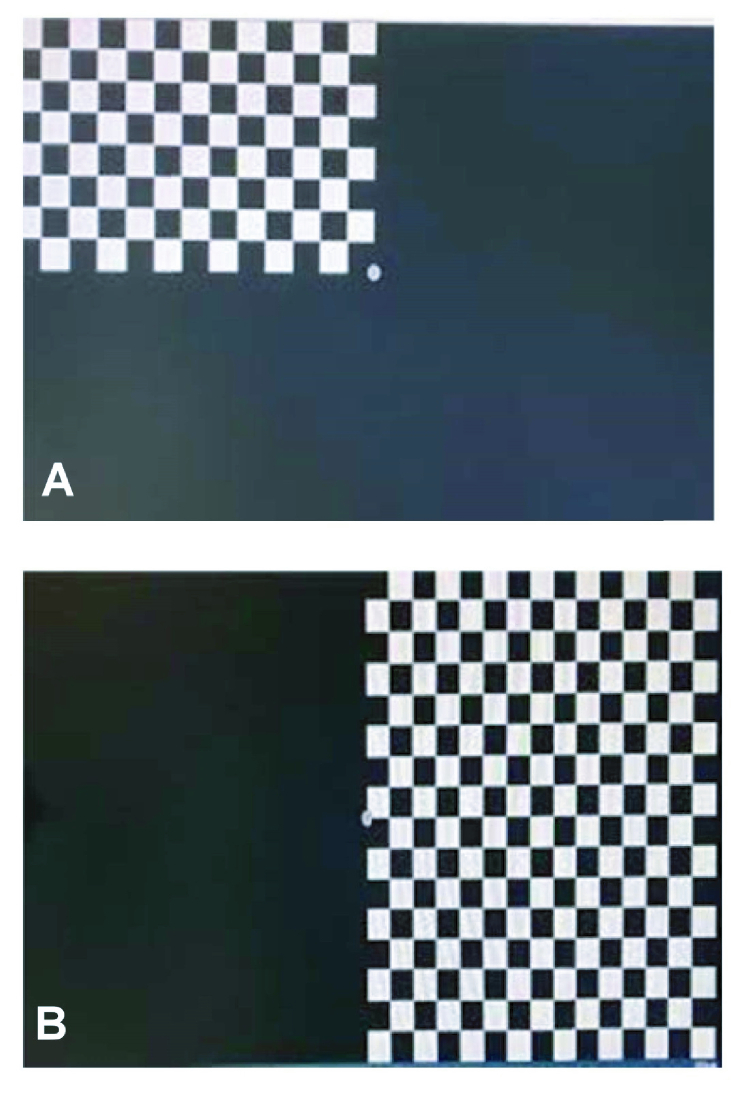
Area pattern (A) and hemifield (B) patterns of visual evoked potential used in this study.

The P100 amplitude and latency of PVEP was assessed and recorded by Metro Vision (Mon pack 3, Perenchies, France) with a check size of 30 min of arc with a contrast of 85%. The test was performed monocularly under mesopic conditions (low-light conditions that are not completely dark) since it is more similar to everyday natural viewing conditions. In this study, the P100 latency and amplitude of the PVEP were measured using passive, active, and ground electrodes. The placement of PVEP electrodes was based on the International 10/20 system.^[12]^ Before the test was conducted, in an effort to achieve better signal transmission, the skin's dead layers were removed with alcohol. The electrodes, which were filled with conductive gel, were then placed on appropriate places on the head and forehead using a special adhesive paste. The resistance of the electrode/skin junction was < 5K Ohms. After recording the patient's name, the patient sat at a distance of 1 m from the stimulation display and the test was performed monocularly. First, a full-field PVEP was recorded for each patient; then, as the patient looked at the fixation point in the middle of the screen, a stimulus was randomly presented at different locations of the screen and the P100 amplitude and latency of PVEP was recorded in the superior-nasal, inferior-nasal, superior-temporal, and inferior-temporal areas as well the superior, inferior, nasal, and temporal areas of the visual field. Each of the visual field areas in the periphery were 30º away from the center. An example of a visual field area is shown in the Figure 1.

The SPSS version 20 was used for statistical analysis. Mean and standard deviation were used to describe the data. According to the Kolmogorov–Smirnov test, all components had a normal distribution. Since the data of different areas within the visual field in each eye was interdependent, repeated measures analysis of variance was used to compare them. A post hoc least significant difference (LSD) test was performed to show comparisons. In this study, a significance level of 0.05 was considered.

Considering that both eyes were analyzed, the correlation effect of fellow eyes was controlled in the analysis.

##  Ethical Considerations

The Ethics Committee of Iran University of Medical Sciences approved the study protocol by the registration number of IR.IUMS. FMN.1395.02, which was conducted in accordance with the tenets of the Helsinki Declaration. All participants signed a written informed consent.

##  RESULTS

In this study, the mean P100 amplitude and latency were assessed in different visual field areas of 80 eyes of 40 patients with a mean age of 23.25 ± 3.44 years.

Of the 40 studied individuals, 20 were women. Table 1 shows the mean and standard deviation of the P100 amplitude and latency in different areas of the visual field. There was no statistically significant difference in the average amplitude and latency of P100 in all areas between males and females (*P* > 0.05).

The repeated measures analysis of variance showed a statistically significant difference among the different areas in terms of amplitude and latency of P100 (*P* = 0.002 and *P* < 0.001, respectively). According to the results, the highest and lowest amplitude of P100 was observed in the inferior-nasal and superior areas, respectively. The highest and lowest latency of P100 was related to the temporal and inferior-nasal areas, respectively. Table 2 shows the comparison of the P100 amplitude and latency in the different areas of the visual field, and the effect size values are also presented. All comparisons were reported using Bonferroni post-hoc test with Bonferroni correction.

**Table 1:** The mean and standard deviation (SD) of P100 amplitude and latency in different areas of visual field.


	**Latency of P100 (ms)**	**Amplitude of P100 (microV)**
**Areas**	**Mean ± SD**	**Mean ± SD**
Full	103.99 ± 6.63	7.95 ± 3.9
Nasal	103.57 ± 12.37	3.89 ± 1.99
Temporal	101.4 ± 7.28	5.19 ± 2.25
Superior	100.22 ± 8.16	4.85 ± 2.82
Inferior	102.48 ± 10.46	3.5 ± 1.76
Superior nasal	100.17 ± 12.95	3.24 ± 1.89
Inferior nasal	109.07 ± 14.52	2.76 ± 1.61
Superior temporal	100.92 ± 8.39	3.9 ± 1.67
Inferior temporal	98.85 ± 8.12	3.22 ± 1.51
	
	
SD, standard deviation; ms, millisecond, microV, microvolts		

**Table 2:** Comparison of P100 amplitude and latency in different areas of visual field.


	**Amplitude of P100 (microV)**		**Latency of P100 (ms)**	
	**Quadrans**	**Effect size (95%CI)**	***P*** -value*	**Effect size (95%CI)**	***P*** -value*
Full	Nasal	4.06 (2.91–5.22)	< 0.001	0.42 (-3.27–4.11)	1.000
	Temporal	2.76 (1.58–3.94)	< 0.001	2.59 (-0.06–5.25)	0.063
	Superior	3.10 (2.15–4.05)	< 0.001	3.77 (1.26–6.28)	< 0.001
	Inferior	4.45 (3.16–5.73)	< 0.001	1.51 (-1.73–4.74)	1.000
	Superior nasal	4.71 (3.39–6.03)	< 0.001	3.82 (-0.55–8.19)	0.177
	Inferior nasal	5.19 (3.68–6.69)	< 0.001	-5.08 (-10.9–0.75)	0.179
	Superior temporal	4.05 (2.82–5.28)	< 0.001	3.07 (0.02–6.12)	0.047
	Inferior temporal	4.73 (3.31–6.15)	< 0.001	5.14 (2.55–7.72)	< 0.001
Nasal	Temporal	-1.31 (-2.22–0.39)	< 0.001	2.17 (-2.35–6.7)	1.000
	Superior	-0.97 (-1.82–0.11)	0.012	3.35 (-0.40–7.10)	0.145
	Inferior	0.38 (-0.48–1.25)	1.000	1.09 (-4.06–6.24)	1.000
	Superior nasal	0.64 (-0.06–1.35)	0.119	3.4 (-0.78–7.58)	0.306
	Inferior nasal	1.12 (0.22–2.02)	0.003	-5.5 (-12.23–1.23)	0.300
	Superior temporal	-0.01 (-0.81–0.79)	1.000	2.65(-1.94 -7.24)	1.000
	Inferior temporal	0.67 (-0.14–1.47)	0.273	4.72 (0.58–8.86)	0.011
Temporal	Superior	0.34 (-0.6–1.28)	1.000	1.18 (-1.96–4.31)	1.000
	Inferior	1.69 (0.82–2.56)	< 0.001	-1.08 (-4.56–2.39)	1.000
	Superior nasal	1.95 (0.94–2.95)	< 0.001	1.23 (-3.40–5.86)	1.000
	Inferior nasal	2.43 (1.41–3.44)	< 0.001	-7.67 (-14.14–1.2)	0.007
	Superior temporal	1.29 (0.59–2.00)	< 0.001	0.48 (-1.87–2.82)	1.000
	Inferior temporal	1.97 (1.08–2.86)	< 0.001	2.55 (-0.80–5.89)	0.491
Superior	Inferior	1.35 (0.33–2.37)	< 0.001	-2.26 (-6.15–1.63)	1.000
	Superior nasal	1.61 (0.60–2.61)	< 0.001	0.05 (-3.72–3.82)	1.000
	Inferior nasal	2.09 (0.91–3.27)	< 0.001	-8.85 (-14.98–2.72)	0.000
	Superior temporal	0.95 (-0.04–1.94)	0.075	-0.7 (-4.31–2.91)	1.000
	Inferior temporal	1.63 (0.55–2.71)	0.000	1.37 (-2.04–4.78)	1.000
Inferior	Superior nasal	0.26 (-0.60–1.12)	1.000	2.31 (-2.59–7.22)	1.000
	Inferior nasal	0.74 (-0.15–1.63)	0.250	-6.59 (-13.10–0.08)	0.044
	Superior temporal	-0.4 (-1.18–0.39)	1.000	1.56 (-2.09–5.21)	1.000
	Inferior temporal	0.28 (-0.45–1.01)	1.000	3.63 (0.17–7.08)	0.029
Superior nasal	Inferior nasal	0.48 (-0.37–1.33)	1.000	-8.9 (-16.05–1.75)	0.003
	Superior temporal	-0.66 (-1.46–0.15)	0.307	-0.75 (-5.61–4.11)	1.000
	Inferior temporal	0.02 (-0.75–0.79)	1.000	1.32 (-3.26–5.89)	1.000
Inferior nasal	Superior temporal	-1.14 (-1.9–0.37)	< 0.001	8.15 (1.25–15.05)	0.007
	Inferior temporal	-0.46 (-1.12–0.20)	0.892	10.22 (4.10–16.33)	< 0.001
Superior temporal	Inferior temporal	0.68 (-0.03–1.39)	0.082	2.07 (-1.51–5.65)	1.000
	
	
*Adjustment for multiple comparisons: Bonferroni CI, confidence interval; ms, millisecond, microV, microvolts					

##  DISCUSSION

According to Tables 1 and 2, there were variances in the recorded latency and amplitude values among different areas within the visual field, which were sometimes significant. Therefore, it seems that within different areas of the retina with corresponding paths, and with the same neurons, the transmission speed of the messages was not the same, and did not have the same destination in the cortex. Some studies also suggest that these differences exist.^[12,13]^ As Silveira points out in her study, the conduction velocity of parvocellular (P) cells is slower than that of magnocellular (M) cells and about 80% of all ganglion cells are parvocellular cells.^[14]^

According to clinical findings and electrophysiological standards, P100 is the most important and stable component of the PVEP.^[12,14]^ Table 1 presents an assessment of P100 latency and amplitude. Considering the importance of P100 latency of PVEP,^[12]^ Table 1 delineates the most important index for the assessment of recorded results. According to Table 1, neurons in the inferior temporal area are the fastest and/or most superficial and/or the closest neurons to active PVEP electrodes. According to studies by Baseler et al, the central visual field which is mainly composed of P cells, sends stimuli to the inferior temporal area of the cortex, and the peripheral visual field, which is mainly caused by M cells, sends stimuli to the posterior area of the parietal cortex.^[15,16]^ According to Horton's findings, macular fibers are generally located in the posterior area of the occipital lobe close to the electrode site, while as we move toward the periphery, the fibers are present in the anterior area of the cortex.^[16,17]^ In addition, according to Holliday and Michael, producers of PVEP responses in the cortex are located at different distances from the active electrode.^[18]^ Also, according to studies performed with functional magnetic resonance imaging (fMRI), fovea is presented in the posterior occipital region and areas with increased eccentricity in the anterior area. The peripheral field is at the back of the parietal cortex and near the junction of the calcarine fissure. The horizontal meridians of the field are in the range of the calcarine fissure and the presentation of the upper part of the vertical meridians is below the calcarine fissure.^[16]^

The findings of the recorded amplitude indicate the difference in neuronal density,^[19]^ change distance recorded,^[20]^ and inhibitory response.^[21]^ According to Table 1, the amplitude corresponding to the inferior temporal area findings was not among high recorded amplitudes.

The reason could be that the density of P and M ganglion cells decreases toward the periphery.^[22,23]^ On the other hand, the recording interval is also effective in the responses. In humans, the visual cortex is projected along the superior and inferior regions of the calcarine fissure. The upper region of the calcarine fissure corresponds to the upper region of the retina where the lower visual field is represented and the upper visual field is represented above the calcarine fissure. According to a study by Jeffroys et al, since the producers of the superior field are under the fissure and further away from the active electrode as compared to the producers of the inferior field, a lower amplitude is expected in the lower half of the retina,^[11,18]^ which is consistent with our findings.

After the inferior temporal area, the fastest or the most superficial response was related to the superior nasal area, followed by the superior and superior temporal areas. The ganglion cells of the superior area of the retina are projected to the superior and medial regions of the lateral geniculate body. These superior fibers are then projected to the posterior region of the parietal lobe^[20]^ and are therefore relatively closer to the active electrode.

The amplitude corresponding to the superior and superior temporal areas was the highest amplitude recorded in the study. This finding may indicate a higher density of ganglion cells in the superior retina. On the other hand, these cells are located above the calcarine fissure and are closer to the active electrode as compared to the inferior area and therefore already possess better amplitude. The superior temporal area had higher amplitude than the superior nasal area. On average, above the horizontal meridian, the temporal retina had the larger response than the nasal retina.^[11]^

The temporal retina had the highest response amplitude, indicating the greater density of nerve fibers in this region.^[16]^ The inferior and nasal retina regions of the retina have a lower amplitude and higher latency. The ganglion cells of the inferior retina are projected to the temporal region of the lateral geniculate body. The ganglion cells of the nasal retina are projected to the contralateral lateral geniculate body in layers 1, 4, and 6; therefore, they are farther away from the ipsilateral active electrode and far from contralateral electrode.^[20]^

On the other hand, in the retinal periphery, the density of ganglion cells is higher in the nasal area, producing a better amplitude than the inferior area.^[22]^ The ganglion cells of the center are more superficial while the ganglion cells of the periphery are deeper.^[24]^ The neurons become deeper and their latency increase as we move from the fovea toward the periphery. Moreover, the inferior nasal had the lowest amplitude in the study. These fibers are projected to the contralateral geniculate body and are farther away from the active electrode.^[20]^ Similar to our study, Min ZHONG's study investigated the topographical changes of the visual field with VEP, which, of course, examined the parameters of the VEP wave in 37 areas of the visual field, with a matrix stimuli including a 61 hexagons pattern. In his study, latency is reduced in the temporal region.^[11]^

Based on the conducted studies, factors such as the position of the electrodes, neuronal density and pathway, and the size of the stimulus affect the electrophysiological responses. Since the majority of the striate cortex responses is related to the central visual field which is presented at the tip of the occipital poles which is closer to the active electrode, a decrease in the responses was seen from the center to the periphery considering the cellular density, distribution of magnocellular and parvocellular neurons, and differences in the velocity of conducting messages to the cortex.

The suggestion for further research is to perform these assessments in patients with glaucoma and other conditions associated with visual field defects and compare the findings with standard perimetry results to determine whether early diagnosis would be possible.

A major limitation of this study is the lack of eye tracking during the PVEP test, hence the possibility of fixation instability and unwanted fine eye movements during the test could affect the accuracy of the results.

In summary, this study evaluated the details of local PVEP distribution in different areas of the visual field. Considering the significant difference in the amplitude and latency of the PVEP in different areas, the inferior temporal (lowest latency) and temporal (highest amplitude) areas have the highest visual sensitivity. These findings are in line with the results of other studies.

##  Financial Support and Sponsorship 

None.

##  Conflicts of Interest

No conflicting relationship exists for any author.
